# A Manual of the Principles of Surgery, Based on Pathology, for Students

**Published:** 1866-08

**Authors:** 


A Manual of the Principles of Surgery, Based on Pathology, for Students. By William Canniff, Licentiate of the Medical Board of Upper Canada; M.D. of the University of New York; M.R.C.S. of England, etc., etc. Philadelphia: Lindsay & Blakiston. 1866.

This is a well-executed octavo volume of 402 pages. As its title and size indicate, it is a manual of surgical pathology, presenting a fair summary of the surgical doctrines of the present time. After the preliminary chapter, on *Nutrition*, the contents of the work are included in five divisions:—

1st. Inflammation, and diseases arising out of inflammation.

2d. The Healing Process, and diseases of the healing process.

3d. External Injuries—Contusions and Wounds.

4th. Diseases of Certain Tissues, Bones, Joints, (including fractures and dislocations,) Arteries, and Veins.

5th. Morbid Growths.

We have not had time to give the work that full reading which is necessary to enable us to speak critically of its merits, but will endeavor to recur to it at a future time.

